# Prevalence, associated risk factors and antimicrobial susceptibility patterns of *Shigella* infections among diarrheic pediatric population attending at Gondar town healthcare institutions, Northwest Ethiopia

**DOI:** 10.1186/s40794-019-0079-7

**Published:** 2019-04-18

**Authors:** Amare Alemu, Mekuanint Geta, Selomon Taye, Setegn Eshetie, Tigist Engda

**Affiliations:** 1 Department of Medical Microbiology, School of Biomedical and Laboratory Sciences, College of Medicine and Health Sciences, University of Gondar, 196 Gondar, Ethiopia; 2 Department of Medical Microbiology, College of Health Sciences, Wachamo University, Wachamo, Ethiopia

**Keywords:** *Shigella*, Prevalence, Risk factors, Pediatric population, Antibiotic resistance, Diarrhea

## Abstract

**Background:**

Diarrhea caused *by Shigella* species remains a major public health threat especially in the pediatric population. A regular surveillance system needs to be in place, in order to explore the burden, antimicrobial resistance patterns and associated risk factors for *Shigella* infections. Therefore, the aim of this study was to assess the prevalence, antimicrobial susceptibility patterns and associated risk factors of *Shigella* infections among diarrheic pediatric population attending at selected healthcare institutions in Gondar town.

**Methods:**

A cross-sectional study was conducted in Gondar town healthcare institutions from January to March 2018. A simple random sampling technique was used to enroll 272 study participants. Structured questionnaires were used to gather socio-demographic, environmental and associated risk factors data. Stool samples were collected from diarrheic pediatric patients and inoculated onto MacConkey media, salmonella-shigella agar, and xylose-lysine deoxy-cholate agar. Identification of the bacterial species was carried out by using biochemical tests. The disc-diffusion method was used to determine the antimicrobial susceptibility of the isolates by standardizing the bacterial suspension with a 0.5 McFarland solution. A statistical analysis was done using SPSS version 20 statistical package and *P*-value < 0.05 was considered as statistically significant.

**Results:**

From the total study participants, 29(10.7%) of the patients were culture positive for *Shigella* species. The habit of eating raw food and nutritional status of children were statistically significant with shigellosis (*p* < 0.05). *Shigella* species were found highly resistant to amoxicillin and tetracycline but susceptible to nitrofurantoin and norfloxacin.

**Conclusion:**

High prevalence of *Shigella* species were detected in this study. Through in-vitro drug susceptibility testing, norfloxacin and nitrofurantoin were found to be effective against the isolates, while high resistance rates were observed for tetracycline, and amoxicillin. The findings highlighted the need for regular drug resistance information for the best management of infections.

## Background

Diarrheal diseases remain one of the most dominant public health problems in these days. Gastrointestinal infections due to *Shigell*a species are important cause of mortality and morbidity worldwide; primarily children under the age of 5 years are at high risk. *Shigella* species are members of *Enterobacteriaceae* characterized by non-lactose fermenters, gram-negative rods, facultative anaerobes, non-motile, and non-gas producer [1, 2].

To initiate infection 100 ingested *Shigella* microorganisms are enough to cause acute diarrhea after 4–7 days [2, 3]. After *Shigella* enter the human body they remain in the cytoplasm of the epithelial cells and spread laterally to invade adjacent cells which result in the formation of abscesses and ulcerations with a high concentration of neutrophils in the stool [1]. *Shigella* species have a lot of virulence factors that allow it to adhere to the epithelium of the intestine, survive stomach acid, invade host cells, evade immune responses, and introduce toxins into the body. Invasion of plasmid antigen B (IpaB) enables it to attach to the host cell and facilitates pathways that destroy macrophages when infection is initiated. Invasion plasmid antigen C (IpaC) triggers proteins to form the actin-polymerizing compound that allows *Shigella* to pass and spread within host cells [1, 4].

*Shigella* species cause mild to severe forms of gastrointestinal tract infections that are limited to the intestinal tract of humans and cause bacillary dysentery which is characterized by watery or bloody diarrhea [1, 3]. Shigellosis is still accounts for a significant proportion of morbidity and mortality especially; in children with diarrhea in developing countries. Globally; Shigellosis causes an approximately of 165 million cases of severe diarrhea [1]. In developing world; More than one million death occur yearly due to infections by *Shigella* species however in Africa, an estimate of 115 people dies of diarrheal diseases every hour is mostly due to shigellosis [3, 5, 6].

Global incidences of *Shigella* species are related to many factors comprising of raw meat consumption, extensive field slaughtering practices, unhygienic food handling practices and unsafe water supply [7, 8]. *Shigella* species can be disseminated from person to person feco-orally. Culture, biochemical tests and routine microscopy must be carried out to diagnose shigellosis. For molecular techniques, the most common methods used in the developed world are the pulsed-field gel electrophoresis (PFGE) and multiple loci sequence typing (MLST) [1–3].

Antibiotic resistance has become a major concern to control diarrhea in children. Increasing antimicrobial resistance has complicated for the selection of antibiotics in treating shigellosis. Over the past decades *Shigella* species showed a persistent increase in antimicrobial resistance to routinely prescribed antimicrobials. In Ethiopia, *Shigella* have been reported to be resistant to first-line antibiotics such as ampicillin, tetracycline, and amoxicillin [9–11]. Therefore, identification of the prevalence, associated risk factors and resistance patterns of *Shigella* species in diarrheal pediatric population is compulsory.

## Methods

### Study area, design and period

Institution based cross-sectional study was conducted at the selected health institution in Gondar town from January 2018 to March 2018. The town is 747 km northwest to Addis Ababa, the capital city of Ethiopia. Based on Federal Democratic Republic of Ethiopia Central Statistical Agency (CSA), population projection of Ethiopia from 2014 to 2017, the town has an estimate of 341,991 populations [12]. One comprehensive specialize referral hospital and 8 health centers and one private specialized pediatric clinic are currently giving health service to the community. Among these, one comprehensive specialized hospital, two health centers and one specialized pediatric clinic are selected for the study.

### Population

The Source population was all pediatric population under 14 years of age and lives in Gondar town during the study period. And the study population was all diarrheic pediatric populations in the selected health institutions seeking medical service during the study period.

### Sample size determination and sampling techniques

The sample size was calculated using a single population proportion formula based on the assumption of 5% expected margins of error, considering 95% confidence interval, 10% non-response rate and by taking the prevalence of 20.1% from the previous study [13]. The final sample size became 272.

Simple random sampling method was employed to select study sites and their sample size was allocated proportionally. Accordingly; 119, 25, 30 and 98 study subjects were enrolled in Gondar university compressive specialized hospital, Maraki health center, Gondar health center and Dr. Mihretie specialized pediatric clinic respectively.

### Data collection and laboratory processing

A structured questionnaire was administered to the child’s parent or guardian to collect data on basic socio-demographic character and potential risk factors for infection. A clean stool cups were given to parents or guardians with proper instruction on specimen collection. Each specimen was checked for its labeling, quantity and procedure of collection. Then the specimens were transported with selenite F broth transport media to University of Gondar medical microbiology laboratory.

### Culture and identification

The collected samples were immediately processed for bacteriological analysis. A loop full stool sample suspension was inoculated onto MacConkey and incubated at 37 °C for 24 h. Pure non-lactose fermenter colony was transferred to salmonella-shigella agar (SS agar) and xylose-lysine-deoxycholate agar (XLD) and incubated at 37 °C for further 24 h. Colorless colonies from MacConkey agar, red-pink colonies from xylose-lysine-deoxycholate agar, and colorless colonies mostly with black centers due to H_2_S production on SS agar were considered non-lactose fermenting colonies. Culture-negative specimens on SS agar and XLD agar media were sub-cultured in Selenite F broth to improve the recovery of the isolates and were identified by repeated sub-culturing before discarded. Typical non-lactose fermenter colonies were subjected to standard biochemical tests to identify *Shigella* species. Biochemical tests were also carried out by inoculation of Klingler iron agar, Simon’s citrate agar, lysine iron agar, and MIU test (motility test, Indole and Urease production) for final identification.

### Antimicrobial susceptibility testing

Suspension of *Shigella* species were prepared by taking pure colonies from isolates with a sterile wire loop and suspended in sterile nutrient broth and then incubated for 2 h [14]. The turbidity of the suspensions to be inoculated were equilibrated to match with 0.5 McFarland standards. A sterile cotton swab was used and additional suspensions were removed by gentle rotation of the swab against the surface of the tube and then spread consistently over the Muller Hinton agar plate. The selected antimicrobials were dispensed using sterile forceps on the medium and incubated at 37 °C for 24 h. Then the diameter of zone of inhibition around the disc was measured to the nearest millimeter using a caliper and the isolates were classified as sensitive, intermediate, and resistant as recommended by “(CLSI 2017)”. Each isolate was tested for amoxicillin (10 μg), tetracycline (30 μg), cotrimoxazole (25 μg), chloramphenicol (10 μg), norfloxacin (5 μg), nitrofurantoin (30 μg), azithromycin (15 μg), and amoxicillin-clavulanate (10 μg) and interpreted as sensitive; intermediate and resistant following the method of CLSI 2017 [15].

### Data management and quality assurance

To generate quality and reliable data; all questions were prepared in a clear and precise way and translated into local language (Amharic). Data collectors were trained and the entire questionnaire was checked for completeness, during and after data collection by the data collectors. The raw data were checked for completeness and representativeness before entry into database. Moreover, all laboratory procedures were done by maintaining quality control procedures by following standard operating procedures (SOPs) in the detection and identification of *Shigella* in stool samples. The culture media were checked for sterility by incubating representative of the batch at 37 °C overnight and observing the growth of organism. Those batches of the media that showed growth were discarded. Control strains of *Escherichia coli* ATCC 25922, Salmonella Typhi ATCC 13311, Salmonella enteritis’s ATCC 13076, Salmonella Sonni ATCC 25331 were used for culture and sensitivity testing [16].

### Data analysis

After the completion of data collection, the questionnaire was checked for its completeness, unrecorded values and unlikely responses and then cleaned up manually on such indication. The laboratory analysis result was registered on the laboratory data collection format sheet. From data collection format sheet, pre-coded and checked data was entered to Epi data version 3.1 and then exported to SPSS version 20 for statistical analysis. The base line characteristics of the study population were summarized using descriptive statistics. Internal comparison was made using logistic regression to determine the effect of independent variables by calculating the strength of association between infection and associated factors using odds ratio (OR) and taking 95% confidence interval (CI). Odds Ratio was computed using binary logistic regression analysis. Adjusted OR with 95% CI were used to assess the strength of association and *P*-value < 0.05 were used to assess statistical significance.

### Ethical consideration

This study was ethically approved by Ethical Review Committee of University of Gondar, College of Medicine and Health Sciences, school of Biomedical and Laboratory Sciences. Permission was sought from each health institution. Written assent was taken from each children’s parent/guardian after they understood the purpose of the study. All the subjects’ data were kept in full confidentiality and was not be disclosed to an unauthorized person.

## Results

### Socio-demographic characteristics

A total of 272 diarrheic pediatric patients of age ≤ 14 years were included in the study. Of these, 15(55.5%) were males and 12(44.5%) were females. The mean age of the participants was 4.6 years. The majority, 18(66.5%) of the study participants were under 5 years. Most of the participants, 25(92.3%) were from the urban residence and 80(29.4%) of the parents completed at least college diploma (Table 1).

**Table 1:** Distributions of socio-demographic characteristics of children and parents attended at selected Gondar town healthcare institutions, Northwest Ethiopia, 2018, *n* = 272

Variables	Categories	Frequency	Percent(%)
Sex	Male	151	55.5
	Female	121	44.5
Age	≤5	181	66.5
	> 5 years	91	33.5
Residence	Urban	251	92.3
	Rural	21	7.7
Income (family)	< 500 birr	19	7
	500–1000 birr	69	25.4
	> 1000 birr	184	67.6
Educational status	Illiterate	59	21.7
	Only read and write	32	11.8
	Primary school	54	19.9
	Secondary school	47	17.3
	College and university	80	29.4

### Prevalence of *Shigella* species

Of the total 272 study participants, 29(10.7%) were positive for *Shigella* species*.* The highest prevalence 22(12.2%) of *Shigella* species were found in under five years children. Seven (7.7%) *Shigella* species were observed in the age group above five years (Table 2).

**Table 2:** Prevalence and associated risk factors of *Shigella* species in diarrheic pediatric population attended at selected Gondar town healthcare institutions, Northwest Ethiopia, 2018, *n* = 272

Variables	Categories	Results of *Shigella* species				
		Positive (%)	Negative (%)	COR(95%CI)	AOR (95%Cl)	P-value
Sex	Male	18(11.9%)	133(88.1%)	1.35(0.61–2.98)	–	
	Female	11(9.1%)	110(90.9%)	1	–	
Age	≤5 years	22(12.2%)	159(87.8%)	1.66(0.68–4.04)	–	
	> 5 years	7(7.7%)	84(92.3%)	1	–	
Breast feeding	Yes	14(12.4%)	99(87.6%)	1	–	
	No	15(9.4%)	144(90.6%)	0.74(0.3401.59)	–	
Residence	Urban	25(10.0%)	226(90.0%)	1	–	
	Rural	4(19.0%)	17(81.0%)	2.13(0.66–6.82)	1.43 (0.35–5.89)	0.622
Income (family)	< 500	4(19.0%)	17(81.0%)	2.80(0.83–9.45)	2.69 (0.62–11.63)	0.186
	500–1000	9(13.4%)	58(86.6%)	1.56(0.66–3.75)	–	
	> 1000	16(8.7%)	168(91.3%)	1	–	
Educational (family)	Illiterate	15(25.4%)	44(74.6%)	5.11 (1.74–15.03)	3.49 (1.01–12.04)	0.047
	Only read and write	1(3.1%)	31(96.9%)	0.48 (0.05–4.31)	–	
	Primary school	6(11.1%)	48(88.9%)	1.87 (0.54–6.48)	–	
	Secondary school	2(4.3%)	45(95.7%)	0.67(0.12–3.58)	–	
	College and university	5(6.2%)	75(93.8%)	1	1	
Water source	Unprotected well water	2 (18.2%)	9 (81.8%)	1.94 (0.40–9.47)	–	
	Surface water	2(11.1%)	16(88.9%)	1.09 (0.24–5.02)	–	
	Piped tap water	25(10.3%)	218(89.7%)	1	–	
Domestic animals	Yes	8(14.3%)	48 (85.7%)	1.55(0.65–3.71)	–	
	No	21(9.7%)	195 (90.3%)	1	–	
Raw food	Yes	21 (18.4%)	93 (81.6%)	4.23 (1.80–9.95)	3.66 (1.45–9.24)	0.006
	No	8(5.1%)	150(94.9%)	1	1	
Toilet	Open defecation	14 (20.9%)	53 (79.1%)	3.35 (1.52–7.37)	1.88 (0.73–4.84)	0.192
	Latrine usage	15(7.3%)	190 (92.7%)	1	–	
Hand Washing	Before preparing food	13 (12.3%)	93 (87.7%)	1.94 (0.66–5.68)	1.45 (0.44–4.73)	0.541
	After defecating child	6 (12.2%)	43 (87.8%)	1.95 (0.56–6.79)	1.50 (0.36–6.19)	0.579
	After toilet use	5 (11.9%)	37 (88.1%)	1.94 (0.53–7.16)	–	
	After cleaning anything	5 (6.7%)	70 (93.3%)	1	–	
Nutritional status	Normal	24(9.3%)	235 (90.7%)	1	1	
	Malnourished	5(38.5%)	8 (61.5%)	6.12 (1.85–20.19)	5.56 (1.42–21.76)	0.014

*COR* Crude Odds Ratio, *AOR* Adjusted Odds Ratio

The distribution of *Shigella* species in Gondar University specialized Hospital, Gondar Health center, Maraki Health center and Dr. Mihretie Specialty Pediatric private clinic were 7(5.9%), 5(16.7%), 6(24.0%) and 11(11.2%) respectively (Table 3).

**Table 3:** The distribution of *Shigella* species in relation to different study sites in diarrheic pediatric population attended at selected Gondar town healthcare institutions, Northwest Ethiopia, 2018, *n* = 272

Bacterial Species	Results	Hospital and Health Centers			
		GUCRH (*n* = 119)	Gondar Health Center (*n* = 30)	Maraki Health Center (*n* = 25)	Dr. Mihretie Pediatric Specialty Clinic (*n* = 98)
Shigella species	Positive *n* (%)	7 (5.9%)	5 (16.7%)	6 (24.0%)	11 (11.2%)
	Negative *n* (%)	112 (94.1%)	25 (83.3%)	19 (76.0%)	87 (88.8%)

### Antimicrobial susceptibility patterns of *shigella* species

In this study from the isolated *Shigella* species 93.1 and 89.7% were resistant to amoxicillin and tetracycline respectively however 75.9 and 93.1% were susceptible to norfloxacin and nitrofurantoin respectively (Table 4). Moreover; 22(76%) *Shigella* isolates were found to be resistant to a combination of three or more routinely prescribed drugs (Table 5).

**Table 4:** Antimicrobial susceptibility patterns of *Shigella* isolates among diarrheic pediatric population attended at selected Gondar town Healthcare institutions, Northwest Ethiopia, 2018

Antibiotic Disc	Shigella species (*n* = 29)		
	S (%)	I (%)	R (%)
Amoxicillin (AMX)	1(3.4%)	1 (3.4%)	27 (93.1%)
Norfloxacin (NOR)	22 (75.9%)	5 (17.2%)	2 (6.9%)
Cotrimoxazole (SXT)	5 (17.2%)	12 (41.4%)	12 (41.4%)
Amoxicillin-clavulanate (AMC)	14 (48.3%)	12 (41.4%)	3 (10.3%)
Chloramphenicol (CAF)	5 (17.2%)	11 (37.9%)	13 (44.8%)
Tetracycline (TE)	2 (6.9%)	1 (3.4%)	26 (89.7%)
Nitrofurantoin (NIT)	27(93.1%)	2 (6.9%)	0 (0.0%)
Azithromycin (AZM)	13 (44.8%)	6 (20.7%)	10 (34.5%)

*S* Susceptible, *I* Intermediate, *R* Resistance

**Table 5:** Multi-Drug Resistance patterns (MDR) of *Shigella* species isolated from diarrheic pediatric population attended at selected Gondar town Healthcare institutions, Northwest Ethiopia, 2018, *n* = 272

Species	Resistance Antibiotics	Number of organisms	group
*Shigella (n = 5)*	AMX	1	R1
	CAF	1	
	TE		
	AZM	1	
	AMC		
*Shigella (n = 6)*	SXT, AMX	1	R2
	AMC, CAF	1	
	TE, AZM	1	
	AZM, NIT	1	
	TE, CAF	1	
	NOR, AMX	1	
*Shigella (n = 11)*	AMX, TE, CAF, AZM	1	R3
	TE, AMC, AZM	1	
	TE, CAF, AZM	1	
	AMX, SXT, AMC, CAF	1	
	TE, AZM, SXT	1	
	AMX, SXT, CAF	1	
	AMX, SXT, AMC	1	
	TE, AZM, SXT	1	
	AMX, TE, AZM, SXT	1	
	TE, AMC, SXT	1	
	AMX, SXT, CAF, TE	1	

*R1* Resistance for one drug, *R2* Resistance for two drugs, ≥R3 Resistance for three or more drug

### Associated risk factors of *Shigella* species infections

Associated risk factors to *Shigella* species infections were analyzed by using binary logistic regression. Many variables were tend to be more associated with developing an infection but did not reach statistical significance. Multivariable logistic regression showed that raw food consumption, malnutritional status of the children and literacy of parents had a statistically significant association with the prevalence of *Shigella* species infections in the pediatric population. Illiterate mothers’ children were 3.5 (*P* = 0.047; AOR = 3.49; CI = 1.01–12.04) times more likely to be infected with *Shigella*. The prevalence of shigellosis was lower among children whose mothers had secondary and above educational statuses.

The study also showed that there is a statistical association between *Shigella* infection and the consumption of raw foodstuffs. Children who had consumed raw food were 3.7 (*P* = 0.006; AOR = 3.66; CI = 1.45–9.24) times more likely to have *Shigella* infection. Moreover; malnourished children were to be greatly infected with *Shigella* species (Table 2).

## Discussion

Intestinal infections due to pathogenic *Enterobacteriaceae* especially *Shigella* species are important causes of morbidity and mortality globally [1, 2]. In this study the total prevalence of *Shigella* species were 29(10.7%) (95%CI = 7.0–14.0%) in all age groups and was in consistent with studies carried out in Bahir Dar, Ethiopia 8.4% [10], Sydney, Australia 8.9% [17] and Brazil 10% [16]. However; it was lower than studies from Unguja Island-Zanzibar 38.5% [5], India 39.6% [18], Nepal 52.2% [19], Saudi Arabia 61.3% [20], Lebanon 30% [21], Iran 40% [22]. The reason is that the investigators might use advanced techniques for the detection of *Shigella* species. In contrast the result was higher than studies carried out from Hawassa, south Ethiopia 3.6% [23], Jimma, southwest Ethiopia 1.1% [9], Ankara, Turkey 3.2% [24], China 3.6% [25], Windhoek, Namibia 1.3% [26]. This might be due to associated risk factors exposed to *Shigella* species differ in different place and countries; as a result, the prevalence of shigellosis would be different in time and place.

The highest prevalence of 22(12.2%; 95% CI = 7.2–17.1%) *Shigella* species in this study were observed in children aged under five years. This result is in agreement with a study carried out in Addis Ababa, Ethiopia (9.1%) [27], Mekelle, northern Ethiopia (13.3%) [28], Windhoek, Namibia, (13.3%) [26], and Sydney, Australia (11.1%) [17]. The main reasons are younger children are exposed to many environmental factors such as playing with contaminated soil, eating contaminated food and drinking contaminated water than an elder child. Furthermore; small children are immunologically naive and delay in humoral immunity response resulted to more susceptible to infection and also they can produce little hydrochloric acid (gastric HCl) which is a natural barrier to many microorganisms [1, 16, 29]. Seven (7.7%)(95% CI = 3.3–13.2%) of *Shigella* species were isolated in children aged above five years which was in agreement with studies done in Bahir Dar, northwest Ethiopia (3.3%) [10] and Sydney, Australia (6.7%) [17].

Multivariable logistic regression showed that raw food consumption, nutritional status of the children and illiteracy of parents has a statistically associated with the prevalence of *Shigella* species infections in pediatric population. In this study, malnourished children were 5.6 (*P* = 0.014) times more likely to be infected with *Shigella* species. This is due to the fact that malnourished children are unable to produce protective immune system which leads to varies enteric disease including shigellosis. Raw food consumption also 3.7(*P* = 0.006) times more likely to develop *Shigella* infection as compared with those who did not eat raw food. This might be due to raw foodstuffs were contaminated with waste products of an infected individuals and consumption of water and food from unprotected sources. Moreover, illiterate mothers (parents) were 3.5(*P* = 0.047) times more likely to expose their children for *Shigella* species infection than any other educated mothers or parents. This might be due to lack of knowledge on risk factors which exposed children to diarrheal diseases.

*Shigella* isolates in this study were susceptible to norfloxacin (75.9%) and nitrofurantoin (93.1%). This was in line with a study done in Bahir Dar town, Ethiopia nitrofurantoin (93.9%) [30] and Kathmandu, Nepal, norfloxacin (91.7%) [19]. However, *Shigella* species in this study were resistant to amoxicillin (93.1%) and tetracycline (89.7%) which was comparable with studies conducted in Harar, Eastern Ethiopia, tetracycline (96.4%) [11], Harar, Eastern Ethiopia, tetracycline (70.6%) [31], China, amoxicillin (93.2%) and tetracycline (90.9%) [32]. Twenty-two (76%) *Shigella* species in this study were resistant to three or more routinely prescribed drugs. The high percentage of resistant isolates to amoxicillin, tetracycline and other drugs might be either they were regularly and unnecessarily prescribed or people can have easily access to these drugs from community pharmacy without prescription [14]. Drug resistance varies in different areas might be due to the difference in strains of *Shigella* species.

## Conclusions

High prevalence of *Shigella* species infections were demonstrated in this study, dominantly in children under five years. Besides the associated risk factors such as the habit of eating raw food and the nutritional status of the child predisposes them to *Shigella* species infections. In addition, high drug resistance in *Shigella* species was observed especially for amoxicillin and tetracycline. This study also revealed that norfloxacin and nitrofurantoin can be used as the drug of choice for shigellosis. Health policy makers and stakeholders must be vigilant to prevent these nasty diseases in pediatric population. The limitation of this study was identification of Serogroup of *Shigella* species was not performed due to financial constraints and lack of advanced technology in the existed facilities. Therefore; regular surveillance on the prevalence, antimicrobial susceptibility and identification of serogroups and serotypes of *Shigella* isolates need to be evaluated.

## Abbreviations

AOR: Adjusted Odds Ratio
ATCC: American Type Culture Collection
CI: Confidence Interval
CLSI: Clinical Laboratory Standards Institute
COR: Crude Odds Ratio
GUCSH: Gondar University Comprehensive Specialized Hospital
MDR: Multi-Drug Resistance
MUAC: Middle Upper Arm Circumference
QC: Quality Control
SPSS: Statistical Package for Social Sciences
SS Agar: Salmonella-Shigella Agar
USA: United States of America
WHO: World Health Organization
XLD: Xylose-Lysine-Deoxycholate Agar
